# A Case of Ovarian Dropsy

**Published:** 1841-10

**Authors:** J. B. Kissam


*A Case of Ovarian Dropsy.* By J. B. Kissam.—Mrs. D., aged 35 years, is the mother of ten children, had enjoyed good health till Feb. 1840, when she discovered an enlargement in the right side of the abdomen, and œdema of the lower limbs; in two months after she ceased to menstruate. Fluctuation being perceptible in the tumor, the use of calomel, squills, and digitalis was determined on. Under this treatment there was a decided diminution of size, and a single return of the monthly secretion. In September, however, the abdominal swelling again showed itself, with extensive anasarca of the lower half of the body, and of the abdomen, so great as to make the fluctuation very obscure. Her general health remained unimpaired, and she only complained of the great weight, difficulty of respiration, and an inability to lie down. The remedies before tried, were again resorted to, without benefit, and the increase of the fluid was so rapid, that paracentesis became necessary early in January last. The puncture was made at the usual point, midway between the umbilicus and pubis. The depth of the adipose and infiltrated cellular tissues was so great (nearly three inches) that after making the external incision with a scalpel, a large trocar was passed entirely to the guard of the canula, and the latter firmly pressed inwards before the fluid could be made to flow. Instead of passing with the usual facility into the sack, the instrument seemed to meet with the resistance of a firm body, which was probably one of the tumors so commonly connected with the cyst in this disease. The fluid oozed so slowly from the wound, that the canula was withdrawn, and the incision left open, that the discharge might thus take place. On the following day it was ascertained that in the course of four hours, there had been evacuated forty-eight pints of a fluid, which soon firmly coagulated, and had precisely the appearance of blood; however, upon compressing a handful of it, it left no sanguineous stain; neither did the pulse give evidence of the system having sustained a loss of the vital fluid.

The abdomen, though somewhat softened, was not apparently much lessened in size. It was evident that the fluid was not entirely discharged; some relief was derived from the operation, and she was able to lie down for five or six weeks, after which a rapid accumulation took place. Medical treatment was resorted to, but without effect, and in the latter part of June it became necessary to repeat the operation. At this time, the anasarcous swelling of the abdomen and lower limbs was enormous, and fluctuation was discoverable only above the umbilicus. The patient could scarcely rise from her chair without assistance, and suffered much from dyspnœa; the measurement around the largest part of the body was *six feet and nine inches*. Owing to circumstances, my friend Dr. Hoffman advised me to make the incision and puncture *above* the umbilicus; from which a free discharge of transparent fluid was obtained, which soon coagulated. The inconvenience met with in the former operation from the depth of the infiltrated cellular tissue was at this time obviated by a suggestion from Prof. Gilman, viz: that the anasarca should be lessened around the point to be punctured, by pressing and kneading the part with the fingers for some minutes. After the perfect evacuation of the sac, the quantity drawn off proved by measurement to be *seventy-nine pints*.

The wound healed kindly, without any unpleasant symptoms. A new mode of treatment was now resorted to. She was put on the steady use of hydrarg., prot. iod., p. scillæ., and p. digital., which, after five weeks slightly affected the gums and acted very freely upon the kidneys. Three months have now elapsed, the patient's general health is very good, the anasarca has entirelp disappeared, and she has once menstruated; being the second time in the period of eighteen months. From a recent examination by several medical friends, it is evident there is no return of the disease, both iliac regions being in a natural condition, and the abdomen perfectly flaccid.

From the previous speedy return of the disease, and the period which has elapsed since the operation, I am led to hope that its re-appearance need not be looked for; and that this may prove one of the few cases which yield to medical and surgical treatment combined.

*Remarks.*—The sanguineous appearance of the fluid first evacuated was new to me and to those who saw it. The resemblance of the fluid to blood was so exact that most of those who saw it, did not hesitate to pronounce it blood. Whence was this color? probably from a small quantity of blood being mingled with the fluid, the blood having flowed from some artery wounded in the operation. The trocar, as I have mentioned, seemed after having penetrated the abdominal wall to pass for some distance through a firm, dense structure. This was probably one of those tumors by which the walls of ovarian cysts are very frequently occupied—in it the blood vessel which supplied the blood, probably existed, and it was the dense fibrous structure of this tumor which prevent the free flow of the fluid on the first operation. That the bloody hue of the fluid was accidental, is proved by the fact that altho' the fluid was not entirely evacuated on the first operation, yet at the second it was found quite clear, though coagulable as before.

*By the Editor of the Medical Gazette.*—We record this case with peculiar satisfaction, because it was successfully treated by a remedy which, if not new, has been but little used. Mercury has, we all know, been frequently tried, but the weight of authority is against it. Iodine too has had its advocates, but we have few proofs, in the shape of well authenticated cases, of its curative powers. It would seem from Dr. Kissam's case, that more may be expected from the two remedies combined in the shape of iodide of mercury than from either singly. We recommend the remedy to the notice of our readers: let it be freely and fairly tried. That the trials should be fair, they will bear in mind that no treatment can be expected to avail, where the dropsical accumulation is the consequence of schirrus of the organ. This state (schirrus) cannot perhaps be certainly diagnosticated, but it should be suspected where the constitution is much broken, where there is evidence of cancerous cachexy, or where schirrus exists or has existed in any other organ. Again, to give this remedy a fair trial, the fluid should be fully evacuated first, and then, the belly being well supported by tight bandages, let the diuretic be given till the gums are touched. The beneficial effect of tight bandaging is strongly, though not too strongly, insisted on by Hamilton; it should never be neglected. We may not dismiss this case of successfully treated ovarian dropsy, without a reference to the various surgical operations, those detestable "*feats of surgical daring*," which have been proposed and tried for the cure of the disease, and which have all been defended by assuming that the disease for which they were proposed was utterly incurable. We have in some editorial hints, on surgical ethics, already expressed our opinion of the propriety of this notion, that a surgeon is justified in trying any and every operation on a patient where she happens to have a disease which he thinks incurable, that is which *he does not know how to cure.* This, as we have said, is no reason at all for operating: the propriety of that step is to be settled by other and far different considerations. But how will the gentlemen who have been in the habit of very formally and, as they fancy, very scientifically, *putting their patients with ovarian disease to death*, whether by excision or by the introduction of tents, trocars or the like, feel, when they find that this so called incurable disease, has yielded to medical treatment after two tappings, one of them, as we believe, the largest on record (seventy-nine pints.) Surely one such case (we do not mean to say it is the only one on record,) should put these barbarous operations out of the mind of every man who means to be anything but a mere barber surgeon. We have no doubt if it had been Mrs. D.'s fate to fall into the hand of one of these gentry, she would now have been "sleeping in a bloody grave," and the records of science would have been disgraced, and the heart of every man of common humanity sickened, by the detail of another "*feat of surgical daring;*" perhaps we should have had the bloody history in all its fearful and disgusting details trumpeted forth in the penny papers, that all the world might know how recklessly Dr. —— could cut and carve the human frame. And then, the butcher would have called himself, and others would have called him, a surgeon. Thank God, such was not the fate of Mrs. D., and as a consequence, she lives, the centre round which the fond affections of a social circle cluster, and the barber surgeons have one the less murder to answer for. We congratulate Dr. Kissam, with our whole heart, on the result of his *case.—N. Y. Med. Gaz.*

